# Cross-Sectional Associations between Home Environmental Factors and Domain-Specific Sedentary Behaviors in Adults: The Moderating Role of Socio-Demographic Variables and BMI

**DOI:** 10.3390/ijerph14111329

**Published:** 2017-10-31

**Authors:** Sofie Compernolle, Cedric Busschaert, Ilse De Bourdeaudhuij, Greet Cardon, Sebastien F. M. Chastin, Jelle Van Cauwenberg, Katrien De Cocker

**Affiliations:** 1 Department of Movement and Sport Sciences, Faculty of Medicine and Health Sciences, Ghent University, 9000 Ghent, Belgium; cedric_busschaert@hotmail.be (C.B.); Ilse.Debourdeaudhuij@ugent.be (I.D.B.); Greet.Cardon@ugent.be (G.C.); sebastien.chastin@gcu.ac.uk (S.F.M.C.); Katrien.decocker@ugent.be (K.D.C.); 2 School of Health and Life Science, Institute of Applied Health Research, Glasgow Caledonian University, Cowcaddens Road, Glasgow G4 0BA, UK; 3 Research Foundation Flanders (FWO), 1000 Brussels, Belgium; jelle.vancauwenberg@ugent.be; 4 Department of Public Health, Faculty of Medicine and Health Sciences, Ghent University, 9000 Ghent, Belgium

**Keywords:** context-specific sitting time, home environment, interaction, weight status

## Abstract

Despite the negative health effects of too much sitting, the majority of adults are too sedentary. To develop effective interventions, insight is needed into home environmental correlates of adults’ sedentary behaviors, and into the susceptibility of population subgroups to these home environmental cues. In total, 559 Flemish adults reported socio-demographics, weight and height, home environmental factors and domain-specific sedentary behaviors. Generalized linear modeling was conducted to examine main associations between home environmental factors and domain-specific sedentary behaviors, and to test the moderating role of socio-demographics and BMI on these associations. In case of significant interactions, stratified analyses were performed. Results showed that, among those who did use a computer/laptop during the last week, a one-unit increase in the number of computers or laptops was associated with 17% (OR = 1.17; 95% CI = 1.02, 1.34) and 24% (OR = 1.24; 95% CI = 1.08, 1.43) more minutes computer time per day, respectively. The proximity of the remote controller (*p* < 0.001) and the number of televisions (*p* = 0.03) were positively associated with television time, and the number of motorized vehicles (95% CI = 0.001, 0.12) was positively associated with the odds of participation in transport-related sitting time. The latter two associations were moderated by BMI, with significant positive associations limited to those not overweight. To conclude, home environmental factors were associated with domain-specific sedentary behaviors, especially in healthy weight adults. If confirmed by longitudinal studies, public health professionals should encourage adults to limit the number of indoor entertainment devices and motorized vehicles.

## 1. Introduction

Reducing sedentary behavior, which can be defined as any waking activity characterized by an energy expenditure ≤1.5 metabolic equivalents while being in a sitting, lying or reclining posture [1], has become an important public health goal worldwide [2]. Recent research using accelerometers has shown that adults (>18 years) spend on average 8.5 h/day sedentary [3,4]. This is alarming given the evidence for a positive association between sedentary behavior and physical and mental health problems [5].

As has been suggested by the ecological model of four domains of sedentary behavior [6], sedentary behavior is influenced by factors at multiple levels—including individual, social, organizational, environmental, and policy level. A recent review of O’Donoghue and colleagues listed the factors related to adults’ sedentary behavior per level, and concluded that consistent associations were mainly found between individual level socio-demographic factors and sedentary behaviors [7]. However, these factors are rarely modifiable, and are therefore less relevant for the design of sedentary behavior interventions [8]. Consequently, efforts should be made to identify modifiable risk factors of sedentary behavior, such as, for example, physical environmental factors.

Previous studies examining associations of physical environmental factors with sedentary behaviors have yielded few conclusive and often contradictory findings. A potential reason might be that the included physical environmental factors were often not domain-specific (i.e., not specifically related to household sedentary behavior, work-related sedentary behavior, leisure time sedentary behavior or transport-related sedentary behavior), and not related to the person’s immediate home environment (i.e., previous associations were mainly examined with neighborhood characteristics). However, individuals receive higher exposure to home environmental cues compared to their neighborhood environment [9]. Especially for sedentary behavior, it is likely that the presence and availability of entertainment devices and motorized vehicles prompt sedentary behavior [9].

As implied by the ecological model [6], and mapped by the recently developed ‘Systems of Sedentary behaviours’ (SOS) framework [10], the influence of home environmental factors on sedentary behavior might differ across population subgroups. More specifically, we expect that home environmental factors will be more important for subgroups that generally spend more time at home. For example, the presence of indoor entertainment devices might be more important for older adults, and for women and those with children, as their age-related declines in functionality and mobility [11], and their greater caregiving responsibilities [12], respectively, make them spend more time at home. Apart from these socio-demographic subgroups, weight status might also moderate the associations between home environmental factors and sedentary behaviors, as overweight and obese people tend to spend more time at home due to their psychological (e.g., higher odds of depression), social (e.g., less close friends) and economic (e.g., higher odds of unemployment) disadvantages compared to their healthy weight counterparts [13,14]. Insight into these and other potential socio-demographic/BMI moderators is important to identify which (risk) groups are most affected by particular home environmental factors [15].

**Figure 1:**
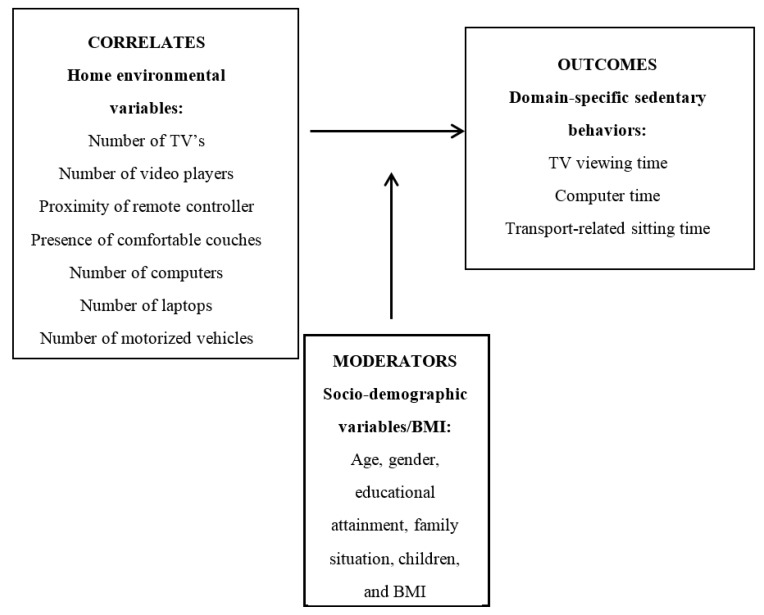
Moderation model: potential socio-demographic/BMI moderators in the relation between home environmental variables and domain-specific sedentary behaviors.

Therefore, the aim of this study was to examine the associations between home environmental correlates and sedentary behaviors (television [TV] time, computer time, and transport-related sitting time) in adults, and to explore the moderating effects of socio-demographic factors and BMI on these associations (see Figure 1).

## 2. Materials and Methods

### 2.1. Study Design

For the present study, cross-sectional survey data were collected as part of the Busschaert study [16,17] to determine correlates of sedentary behaviors in adults (25–60 years) and older adults (≥65 years).

### 2.2. Recruitment and Participants

The public service of Sint-Niklaas (i.e., city in Flanders, Belgium) randomly selected 1917 adults (25–60 years), and 961 older adults (≥65 years) from the municipal register. The recruitment process within this selected sample differed for the two age groups. Selected adults received an information letter and a paper-based questionnaire by regular mail in April 2013. Three weeks later, a reminder was sent. In total, 331 adults filled out the questionnaire (response rate: 17.5%), of which 30 adults were excluded because: their partner filled out the questionnaire (*n* = 21), they were not able to stand (*n* = 7), or they returned the questionnaire after the deadline was exceeded (*n* = 2). This resulted in a final sample of 301 adults (response rate: 15.8%). The older adults received an information letter by regular mail in September 2013. As older adults may experience cognitive difficulties when responding to a paper-based questionnaire [18], they were contacted by telephone up to three times to make an appointment for a structured questionnaire interview at home. In total, 860 older adults could be reached by telephone, of which 293 agreed to participate (response rate: 30.5%). Of these, 35 older adults were excluded because: they were not able to participate due to illness (*n* = 30), they did not speak Dutch (*n* = 4), or they were not able to stand (*n* = 1). This resulted in a final sample of 258 older adults (response rate: 28.1%), who completed a face-to-face interview at home taken by different researchers. All researchers were thoroughly trained by the main researcher (i.e., an information session was organized on conducting the interviews, and all researchers were assisted during their first interview). Participants’ answers were written down by the interviewer on a paper-based questionnaire. All study participants provided written informed consent. The study protocol was approved by the Ethics Committee of the Ghent University Hospital (B670201317406).

### 2.3. Measures

All measures were collected using age-specific questionnaires developed by Busschaert et al. for assessing domain-specific sedentary behaviors and correlates in adults and older adults [19].

**Home environmental factors.** The assessed home environmental factors varied by domain-specific sedentary behavior. For TV viewing time, the number of TVs and the number of video players at home were asked, as well as the degree of agreement with the following statements ‘The remote controller (TV) can always be found close to me when I need it’ (further referred to as ‘Proximity of remote controller’), and ‘The couches at our place are comfortable to sit for a long time’ (further referred to as ‘Comfortable couches’). Both statements were rated using a five-point Likert scale ranging from totally disagree to totally agree. For computer time, the number of computers, and the number of laptops at home were asked. For transport-related sitting time, the number of motorized vehicles in the household was asked. Reliability scores of the above-mentioned items can be found in Supplementary Materials.

**Domain-specific sedentary behaviors.** Domain-specific sedentary behaviors were assessed by the following questions: During the last seven days, how much time did you spend sitting on an average day (1) while watching TV during leisure time; (2) while using a computer during leisure time; (3) while commuting; and (4) while moving from one place to another during leisure time. All domain-specific sedentary behaviors were asked separately for weekdays and weekend days, except sitting while commuting, which was asked for an average working day. As the vast majority of older Flemish adults aged 65 years and older are retired, sitting while commuting was not asked among older adults. Answer options ranged from zero minutes a day to more than seven hours a day. Each domain-specific sedentary behavior (during leisure time) on an average day was calculated by summing domain-specific sedentary behavior on a weekday, multiplied by five, and on a weekend day, multiplied by two, and by dividing this total by seven. For adults, transport-related sitting time was calculated by summing the time spend sitting while commuting, with the time spend sitting while moving from one place to another during leisure time. Test-retest reliability for domain-specific sedentary behaviors on an average day was excellent for computer use and commuting (ICC ranging from 0.83 to 0.95); good for TV viewing (ICC ranging from 0.63 to 0.82) and moderate for moving from one place to another during leisure time (ICC ranging from 0.22 to 0.73) (see Supplementary Materials) [19]. Validity for total sedentary behavior on an average day was moderate-to-good (Spearman’s ρ = 0.49 in adults, and 0.48 in older adults) [19].

**Socio-demographic variables and BMI.** Potential moderators included age, gender, family situation (single, partner but living apart, married or living with partner, widow/widower), having children (yes, no), educational attainment (primary school, art secondary education, vocational secondary education, technical secondary education, general secondary education, higher education [non-university], higher education [university]), and BMI. For the moderation analyses, age was dichotomized into adults (18–60 years) and older adults (≥65 years); family situation was dichotomized into living with or without a partner; educational attainment was dichotomized into having completed tertiary education, or not having completed tertiary education; and BMI was dichotomized into healthy weight (BMI < 25 kg/m^2^) and overweight/obese (BMI ≥ 25 kg/m^2^).

### 2.4. Statistical Analyses

Statistical analyses were performed using R version 3.1.2 (R Foundation for Statistical Computing, Vienna, Austria), and level of significance was set at *p* < 0.05. Descriptive statistics were calculated separately for adults and older adults, as well as for the total sample. Generalized linear models were used to examine the associations between home environmental factors and domain-specific sedentary time as the Kolmogorov-Smirnov test revealed non-normal distributions of some dependent variables (i.e., computer time and transport-related sitting time). Variance inflation factors between the predictors never exceeded seven, and most were substantially less, indicating that no multicollinearity was present [20]. TV time was normally distributed and a generalized linear model with Gaussian variance and identity link function provided the best model fit based on Akaike’s Information Criterion (AIC). As computer time, and transport-related sitting time were positively skewed and contained a large number of zero counts, zero-inflated negative binomial (ZINB) models yielded the best model fit based on the Vuong test (the Vuong test showed that a zero-inflated negative binomial model was preferred over a negative binomial model) and the AIC (the AIC showed that a zero-inflated negative binomial model was preferred over a zero-inflated poisson model). ZINB models examined the associations with the odds of non-participation in computer time/transport-related sitting time. Simultaneously, among those who did use a computer/sit for transport during the last week, ZINB models examined the association with daily minutes of computer use/transport-related sitting time. Thus, one ZINB model might yield two regression coefficients for each independent variable: an odds ratio (OR) (for the association between the independent variable and the odds of not using a computer/sitting for transport) and a negative-binomial model regression coefficient (representing the proportional changes in minutes/day using a computer/sitting for transport with a one-unit increase in the independent variable for those who did use a computer/sit for transport).

Firstly, basic generalized linear models were built for each sedentary behavior including relevant environmental correlates to examine the main associations. Secondly, the cross-product terms of socio-demographic factors/BMI with home environmental factors were added to the basic models one by one to test the moderating effects of socio-demographic factors and BMI on the association between home environmental factors and domain-specific sedentary behaviors. Finally, when significant interactions were identified, stratified models were fitted for each subgroup. All analyses were adjusted for age, gender, educational attainment, number of children, family situation and BMI.

## 3. Results

### 3.1. Sample Characteristics

Sample characteristics from both adults (18–60 years) and older adults (>65 years) are presented in Table 1. Mean age of the total sample was 57.5 years (SD = 17.7), and just over half of the total sample was female, the majority was married or living with a partner, lower educated and had children. Mean TV time was 162.2 min/day (SD = 93.5). Median computer time was 22.5 min/day (Q1, Q3 = 0.00, 7.14) and median transport-related sitting time was 32.14 (Q1, Q3 = 15.00, 75.00) min/day.

### 3.2. Main Associations of Home Environmental Factors and Domain-Specific Sedentary Behaviors and Moderating Effects of Socio-Demographic Variables and BMI

The results of the main associations and moderating effects are presented in Table 2. Results will be explained below per domain-specific sedentary behavior as categorized by the taxonomy of Chastin et al. [21].

**Television time.** The Gaussian model showed that the number of TVs, and the proximity of the remote controller were positively associated with TV time. For example, one additional TV was associated with 11.97 (SE = 5.59) more minutes TV time per day. Moderation analysis showed that the association between the number of TVs and TV time was affected by BMI. The number of TVs was only significantly associated with TV time in adults with a healthy BMI (see Table 3). Having a one-unit increase in the number of TVs was associated with an increase of 26.37 (SE = 8.27) minutes TV time per day for those with a healthy BMI.

**Computer time.** The logit model with computer time as dependent variable showed that a one-unit increase in the number of computers at home was associated with having a 99.8% lower odds of non-participation in computer time (OR = 0.002; 95% CI = 0.001, 0.013) and the number of laptops at home was associated with having a 99.6% lower odds of non-participation in computer time (OR = 0.004; 95% CI = 0.001, 0.014). In other words, adults with one additional computer at home had 500 (1/0.002) times higher odds of having used a computer/laptop, and adults with one additional laptop at home had 250 (1/0.004) times higher odds of having used a computer/laptop. The negative binomial model showed that, among those who did use a computer/laptop during the last week, a one-unit increase in the number of computers or laptops was associated with 17% (OR = 1.17; 95% CI = 1.02, 1.34) and 24% (OR = 1.24; 95% CI = 1.08, 1.43) more minutes computer time per day, respectively. No moderating effects of socio-demographics and BMI were found for computer time.

**Transport-related sitting time.** The logit model with transport-related sitting time as dependent variable showed that a one-unit increase in the number of motorized vehicles was associated with having 99.0% lower odds of non-participation in transport-related sitting time (OR = 0.01; 95% CI = 0.001, 0.12). In other words, adults with one more motorized vehicle in the household had 100 (1/0.01) times higher odds of having participated in transport-related sitting time. The negative binomial model showed that the association between the number of motorized vehicles in the neighborhood and transport-related sitting time among those who did sit for transport during the last week was moderated by BMI. Only transport-related sitting time of healthy weight people was affected by the number of motorized vehicles. BMI-stratified models showed that for healthy weight people, a one-unit increase in the number of motorized vehicles was associated with 11% (OR = 1.11; 95% CI = 1.00, 1.23) more minutes transport-relates sitting time per day among those who did sit for transport during the last week.

Socio-demographic factors did not moderate any of the associations between home environmental factors and domain-specific sedentary behaviors.

## 4. Discussion

The current study aims to understand (1) whether home environmental factors are related to (older) adults’ domain-specific sedentary behaviors; and (2) whether these associations are moderated by socio-demographic variables and BMI. In contrast to the findings of the earlier studies of Busschaert et al. [16,17], our regression analyses revealed multiple significant associations between the included home environmental factors and domain-specific sedentary behaviors, especially in healthy weight adults. Nevertheless, the direction of the associations remains unclear as the included environmental factors are related to adults’ immediate environment, and adults are having control over it. Adults with a higher exposure to indoor entertainment devices and motorized vehicles might display more sedentary behavior; however, it is also possible that adults who enjoy sedentary activities are more likely to purchase (more) indoor entertainment devices and motorized vehicles. In the latter case, targeting psychosocial factors might be more useful than focusing on environmental changes.

For TV time, two significant positive correlates were found, namely the number of TVs and the proximity of the remote controller. The number of TVs was the strongest correlate of TV time. This finding suggest that adults with more TVs at home, will be more likely to watch television. However, reverse causality is also possible. As such, it might be that adults will acquire more TVs to suit their preference for watching TV. Previous studies examining the association between the number of TVs and the amount of TV time showed mixed results. In the study of Van Dyck et al., no association was detected [22], whereas in the study of De Cocker et al. [23] and the study of Gorin et al. [24], a positive significant association was found. Important to note is that Gorin et al. found only a significant association in the healthy weight group (i.e., BMI < 25 kg/m^2^) [24]. This is in line with our results, as current stratified analyses also showed that the positive association between the number of televisions and television time was limited to those without overweight. As, to our knowledge, no previous studies have examined the association of the proximity of the remote controller with TV time, it is difficult to compare the present results with previous findings.

For computer time, both the number of computers and the number of laptops were significant positive correlates of computer time, suggesting that adults with more computer and laptops at home will be more likely to spend computer time. This corresponds with the finding of Van Dyck et al., who also identified the number of computers as a significant correlate of leisure-time Internet use [22]. Again longitudinal studies are needed to confirm the direction of the association, as it is also possible that adults who make the investment to purchase more computers/laptops enjoy using them so much that changing the environment may not alter their sedentary behavior. The absence of moderating effects on the associations with computer time suggests that the number of computers and laptops is equally important for different subgroups. Importantly, the moderating effect of occupational status was not tested due to insufficient power.

Finally, the number of motorized vehicles was negatively associated with the odds of non-participation in transport-related sitting time. This is not surprising as previous research has indicated that the vast majority of transport-related sitting time exists of sitting in a car [25,26]. Stratified analyses showed that the association only applies in healthy weight adults. More research is needed to confirm this result, and to draw definite conclusions on the direction of the association, as adults in favor of car use might have been more prone to buy a car.

Highly remarkable, and in contrast to our expectations, are the lack of moderating effects of socio-demographic factors. Only BMI significantly affected the associations between home environmental factors and sedentary behaviors, in the way that the significant positive associations were limited to those without overweight. This is in contrast to our expectations, as we speculated that overweight/obese adults would be more affected by the home environment, because they tend to spend more time at home considering their psychological (e.g., higher odds of depression), social (e.g., less close friends) and economic (e.g., higher odds of unemployment) disadvantages compared to their healthy weight counterparts [13,14]. As current results suggest the opposite, it seems that healthy weight adults might be more susceptible to home environmental cues, and will thus probably be more affected by future home environmental interventions to reduce sedentary behavior compared to their overweight/obese counterparts. Unfortunately, the prevalence of sedentary behavior is especially alarming in the latter group [27]. Post hoc analyses indicated that overweight/obese adults generally sit more independent of their environment (data not shown). Although the underlying mechanisms for the higher levels of sedentary behavior were not studied in this study, the increased levels of depression [14], lower levels of self-esteem [28] and the impaired quality-of-life [29] of overweight/obese people might provide an explanation. Hence, more research is needed to explicitly understand factors underlying sedentary behavior in risk groups, such overweight and obese people.

A first strength of this study is the use of reliable questionnaires to assess potential correlates of domain-specific sedentary behavior [19]. Moreover, the older adult questionnaires were conducted through face-to-face interviews, as older adults may experience cognitive difficulties when responding to a paper-based questionnaire [18]. In this way, more precise answers could be obtained as the interviewer could provide additional information if necessary. Another strength of this study was the inclusion of domain-specific environmental correlates of sedentary behaviors. Most previous studies have examined more general correlates, although it has been hypothesized that different domains of sedentary behavior are likely to have distinct correlates [6]. Moreover, the included correlates pertain to the very proximal and immediate home environment of the participants which was deemed a priority for research in the SOS framework [10].

Limitations of the current study include the cross-sectional study design, which does not allow for establishing causal relations. Secondly, the relatively low response rate of our study may have led to selection bias. Although there is a good representation of men (44%) and women (56%), and lower (46.5%) and higher (53.5%) educated individuals, as well as younger (from age 25 years) and older (up to age 99 years) adults [30], it remains plausible that those who are more concerned with their health were more likely to have completed the questionnaire. Finally, no objective measurements of domain-specific sedentary behaviors were used, whereby social desirability bias could not be excluded [31].

## 5. Conclusions

To conclude, preliminary evidence was found for associations between home environmental factors and domain-specific sedentary behaviors, especially in healthy weight adults. If further longitudinal studies can confirm these results, future public health messages should encourage adults to limit the number of indoor entertainment devices and motorized vehicles in order to reduce sedentary behavior.

## Figures and Tables

**Table 1:** Sample characteristics from the study population.

Sample Characteristics	Total (*n* = 559)	Adults (*n* = 301)	Older Adults (*n* = 258)
**Socio-demographic variables and BMI**			
**Age**: years, mean (SD)	57.5 (17.7)	43.3 (24.6)	74.0 (6.2)
**Gender**: %			
Male	46.3	45.5	47.3
Female	53.7	54.5	52.7
**Family situation**: %			
Single	11.9	12.2	11.7
Partner but living apart	4.9	7.0	2.3
Married/living with partner	73.8	79.0	67.7
Widow/widower	9.4	1.7	18.3
**Occupational status** ^: %			
Full-time job	-	71.9	-
Part-time job	-	17.1	-
Household	-	5.4	-
Unemployed/job-applicant	-	2.7	-
Career interruption	-	1.0	-
Retired	-	1.0	-
Student	-	1.0	-
**High educational level** *: %	39.4	52.2	24.3
**Having children**: %			
Yes	80.3	71.6	90.3
No	19.7	28.4	9.7
**Body mass index**: kg/m^2^, mean (SD)	26.1 (4.0)	24.6 (3.5)	27.8 (4.0)
**Home environmental factors**			
Number of TVs, mean (SD)	1.32 (0.74)	1.35 (0.88)	1.29 (0.54)
Number of video players, mean (SD)	1.47 (0.91)	2.05 (0.70)	0.80 (0.62)
Proximity of remote controller, median (Q1, Q3)	5.00 (4.00, 5.00)	4.00 (3.00, 5.00)	5.00 (5.00, 5.00)
Presence of comfortable couches, median (Q1, Q3)	5.00 (4.00, 5.00)	4.00 (4.00, 5.00)	5.00 (5.00, 5.00)
Number of computers, median (Q1, Q3)	1.00 (0.00, 1.00)	1.00 (0.00, 1.00)	0.00 (0.00, 1.00)
Number of laptops, median (Q1, Q3)	1.00 (0.00, 1.00)	1.00 (0.00, 1.00)	0.00 (0.00, 1.00)
Number of motorized vehicles, mean (SD)	1.41 (0.92)	1.76 (1.02)	1.01 (0.54)
**Domain-specific sedentary behaviors**			
**TV time**: min/day, mean (SD)	162.2 (93.5)	129.0 (74.6)	201.0 (98.3)
**Computer time**: min/day, median (Q1, Q3)	22.5 (0.00, 77.14)	38.57 (11.79, 90.00)	7.50 (0.00, 70.71)
**Transport-related sitting time**: min/day, median (Q1, Q3)	32.14 (15.00, 75.00)	53.57 (23.57, 101,25)	22.50 (7.50, 37.50)

^ Occupational status was only asked in adults, as most older adults are retired; * Completed college or university.

**Table 2:** Main associations between home environmental factors and domain-specific sedentary behaviors and moderating effects.

Outcome Variable	Correlates	Model	b (SE) ^1^/OR ^2^/Exp. b ^3^	*p* or 95% CI ^4^	Moderator	*p* ^5^
TV time	Number of TVs	Gaussian	**11.97 (5.59)**	**0.03**	BMI	**0.02**
	Number of video players	Gaussian	−2.83 (5.68)	0.62	-	-
	Proximity of remote controller	Gaussian	**14.80 (3.42)**	**<0.001**	-	-
	Presence of comfortable couches	Gaussian	7.31 (4.08)	0.07	-	-
Computer time	Number of computers	ZINB: logit	**0.002**	**0.001, 0.013**	-	-
		ZINB: negative binomial	**1.17**	**1.02, 1.34**	-	-
	Number of laptops	ZINB: logit	**0.004**	**0.001, 0.014**	-	-
		ZINB: negative binomial	**1.24**	**1.08, 1.43**	-	-
Transport-related sitting time	Number of motorized vehicles	ZINB: logit	**0.01**	**0.001, 0.12**	-	-
		ZINB: negative binomial	1.10	1.00, 1.22	BMI	**0.03**

SE = standard error; OR = odds ratio; Exp. b = antilogarithm of regression coefficient; 95% CI = 95% confidence interval; ZINB = zero-inflated negative binomial. Significant associations are presented in bold. ^1^ b-values were used in the Gaussian model, and represent the increase in minutes/day, with a one-unit increase in the predictor. ^2^ OR’s were used in the logit model. The logit model estimates the associations between the home environmental factors and the odds of non-participation in computer time/transport-related sitting time during the last week. ^3^ Exp. b’s were used in the negative binomial model. The negative binomial model estimate the associations between the home environmental factors and the time spent sedentary for those who did use a computer/sit for transport during the last week. The exponent of the b’s represent the proportional increase in minutes/day using a computer/sitting for transport during the last week with a one-unit increase in the predictor. ^4^ *p*-values were reported in case of a Gaussian model, 95% confidence intervals were reported in case of a ZINB. ^5^ *p*-value of the interaction effect.

**Table 3:** Socio-demographic and BMI stratified associations between home environmental factors and domain-specific sedentary behaviors.

Outcome Variable	Association	Model	Stratified Analyses		
			Groups	b (SE) ^1^/OR ^2^/Exp. b ^3^	*p* or 95% CI ^4^
TV time	Number of TVs * BMI	Gaussian	Healthy weight	**26.37 (8.27)**	**<0.001**
			Overweight/obese	0.82 (7.31)	0.91
Transport-related sitting time	Number of motorized vehicles * BMI	Negative binomial	Healthy weight	**1.11**	**1.00, 1.23**
			Overweight/obese	0.96	0.82, 1.12

OR = odds ratio; 95% CI = 95% confidence interval; Exp. b = antilogarithm of regression coefficient. Significant associations are presented in bold. ^1^ b-values were used in the Gaussian model, and represent the increase in in minutes/day, with a one-unit increase in the predictor. ^2^ ORs were used in the logit model. The logit model estimates the associations between the home environmental factors and the odds of non-participation in computer time/transport-related sitting time during the last week. ^3^ Exp. b’s were used in the negative binomial model. The negative binomial model estimate the associations between the home environmental factors and the time spent sedentary for those who did use a computer/sit for transport during the last week. The exponent of the b’s represent the proportional increase in minutes/day using a computer/sitting for transport during the last week with a one-unit increase in the predictor. ^4^ *p*-values were reported in case of a Gaussian model, 95% confidence intervals were reported in case of a ZINB.

## Supplementary Materials

The following are available online at www.mdpi.com/1660-4601/14/11/1329/s1, Table S1: Test-retest reliability of home environmental factors and domain-specific sedentary behaviors, Data S1: Raw data.

## Abbreviations

BMI	body mass index
SOS	systems of sedentary behaviours
TV	television
AIC	Akaike’s information criterion
ZINB	zero-inflated negative binomial
OR	odds ratio
SD	standard deviation
Q1–Q3	quartile 1–quartile 3
CI	confidence interval
