# Supplementary material for: Cross-Sectional Associations between Home Environmental Factors and Domain-Specific Sedentary Behaviors in Adults: The Moderating Role of Socio-Demographic Variables and BMI
Source: Int J Environ Res Public Health. 2017 Oct 31;14(11):1329. doi: 10.3390/ijerph14111329 (PMC5707968; doi:10.3390/ijerph14111329)
Supplement: Supplementary file 1 [file ijerph-14-01329-s001.zip › ijerph-Table S1-suppl.pdf]

International Journal of
Environmental Research and Public Health

MDPI

Supplementary Materials: Cross-Sectional Associations between Home Environmental Factors and Domain-Specific Sedentary Behaviors in Adults: The Moderating Role of Socio-Demographic Variables and BMI

Sofie Compernolle [1,*], Cedric Busschaert [1], Ilse De Bourdeaudhuij [1], Greet Cardon [1], Sebastien F. M. Chastin [1,2], Jelle Van Cauwenberg [3,4] and Katrien De Cocker [1,3]

Table S1. Test-retest reliability of home environmental factors and domain-specific sedentary behaviors.

Item	Test-Retest Reliability Adults ICC (95% CI)	Test-Retest Reliability Older Adults ICC (95% CI)
**Physical environmental factors**		
Number of TV's (pay TV)	0.86 (0.69, 0.94)	1.00 (1.00, 1.00)*
Number of TV's (no pay TV)	0.77 (0.52, 0.90)	
Number of DVD/video players	0.86 (0.69, 0.94)	0.92 (0.82, 0.97)
Proximity of remote controller	0.50 (0.11, 0.76)	0.11 (−0.34, 0.52)
Presence of comfortable couches	0.56 (0.19, 0.79)	−0.07(−0.49, 0.37)
Number of computers	0.76 (0.50, 0.89)	0.80 (0.56, 0.92)
Number of laptops	0.29 (-0.14, 0.63)	1.00 (1.00, 1.00)
Number of motorized vehicles	0.92 (0.80, 0.97)	1.00 (1.00, 1.00)
**Domain-specific sedentary behaviors**		
TV per weekday	0.77 (0.50, 0.90)	0.82 (0.60, 0.93)
TV per weekend day	0.73 (0.44, 0.88)	0.63 (0.27, 0.84)
Computer per weekday	0.89 (0.75, 0.95)	0.83 (0.58, 0.94)
Computer per weekend day	0.90 (0.76, 0.96)	0.83 (0.59, 0.94)
Motorized transport to go to work	0.95 (0.88, 0.98)	-
Motorized transport during leisure per week day	0.73 (0.45, 0.88)	0.48 (0.06, 0.76)
Motorized transport during leisure per weekend day	0.22 (−0.24, 0.61)	0.71 (0.39, 0.87)

* The number of TV's was asked using one single item in older adults.

© 2017 by the authors. Submitted for possible open access publication under the terms and conditions of the Creative Commons Attribution (CC BY) license (http://creativecommons.org/licenses/by/4.0/).
